# Immune microenvironment-dependent effects of age-associated *Bifidobacterium* strains on gut immunity and microbial diversity

**DOI:** 10.3389/fcimb.2025.1639178

**Published:** 2025-09-16

**Authors:** Yaqin He, Furui Zhang, Zhiqiang Tian, Ruyi Li, Ming Su, Liping Hong, Jun Wen, Cao Zhang, Jinhai Tian, Le Guo

**Affiliations:** 1 General Hospital of Ningxia Medical University, Yinchuan, Ningxia, China; 2 School of Laboratory Medicine, Ningxia Medical University, Yinchuan, Ningxia, China; 3 Department of Oncology II, General Hospital of Ningxia Medical University, Yinchuan, Ningxia, China; 4 Department of Gastrointestinal Surgery Affiliated Hospital of Ningxia Medical University, Yinchuan, China; 5 Medical Science Research Institute, General Hospital of Ningxia Medical University, Yinchuan, China

**Keywords:** γδ T, *Bifidobacterium longum subsp*., *infantis*, *Bifidobacterium adolescentis*, DSS, gut microbial

## Abstract

**Background:**

The applications of probiotics in food and infant formula are greatly increased. *Bifidobacterium*, a genus of beneficial bacteria, plays a crucial role in the human gut microbiota. Despite extensive research on probiotics, how age-associated *Bifidobacteria* strains modulate gut immunity and microbial diversity remains unclear.

**Methods:**

Our present study investigates the immunomodulatory effects of two *Bifidobacterium* strains, *Bifidobacterium adolescentis* (BA) and *Bifidobacterium longum subsp. infantis* (BI), on gut immunity and microbial diversity using three models: a DSS-induced chronic colitis mouse model, germ-free mouse model, and in vitro human intestinal γδ T cell co-culture system.

**Results:**

Transcriptomic analysis in the DSS-induced colitis model revealed differential gene expression, particularly in cytokine signaling pathways and γ-chain-related cytokines crucial for γδ T cell function. Both BA and BI reduced γδ T cell infiltration in colorectal tissues, and modulated immune activation markers, with distinct effects on peripheral blood γδ T cell levels. RNA-seq analysis post-probiotic treatment highlighted strain-specific changes, with BA activating NOD2-like receptor signaling and BI enhancing IL-17 and TNF signaling pathways. Direct co-culture experiments demonstrated BI's robust activation of γδ T cells, while BA showed minimal direct effects. Multi-omics correlation analysis suggested that BA and BI modulated immune responses through microenvironment-dependent mechanisms, offering potential therapeutic insights for gut-related inflammatory diseases.

**Conclusions:**

Our findings provide a theoretical basis for the development of age-associated probiotic intervention strategies, offering new insights into personalized microbiota modulation to enhance immune health and gut homeostasis across different life stages.

## Introduction

The applications of probiotics/prebiotics in food and infant formula are greatly increased (Ke et al., 2021; Lemoine et al., 2023). Adding probiotics/prebiotics to food and infant formula promotes beneficial gut flora growth, inhibit spoilage bacteria, and enhance immune function (Eor et al., 2023; Zhou et al., 2024). The use of probiotics/prebiotics as therapeutic and interventional strategies for clinical diseases has emerged a significant area of research. Randomized controlled trials assess the safety, tolerability, and protective effects against diarrhea of infant formulas containing probiotics or symbiotic (Piloquet et al., 2024). *Bifidobacterium* is a key component of the gut microbiota and plays a critical role in maintaining intestinal health (Hidalgo-Cantabrana et al., 2017). Studies have shown that *Bifidobacterium* helps balance gut microbiota by inhibiting the growth of harmful bacteria, promoting the growth of beneficial bacteria, and strengthening the intestinal barrier function, which reduces the risk of infections and inflammation in the gut (Hou et al., 2022; Yoo et al., 2024). *Bifidobacterium* plays a critical role in reducing gut inflammation and improving gut barrier function. It helps restore microbial balance by inhibiting harmful microbes and promoting the growth of beneficial bacteria, which can mitigate chronic gut inflammation. *Bifidobacterium* reduces chronic gut inflammation, especially in IBD patients, through the production of short-chain fatty acids (Yao et al., 2021; Shin et al., 2023).

Many researchers have focused on the differences in gut microbiota composition at different life stages (Ghosh et al., 2022). Infants are born with nearly sterile gut microbiota, and it begins to develop after birth with breast milk or formula feeding. Research has shown that breastfed infants have a gut microbiota dominated by *Bifidobacterium* and *Lactobacillus*, while formula-fed infants have more *Escherichia coli* and *Bacteroides* (Martin-Pelaez et al., 2022; Pantazi et al., 2023). During childhood, the gut microbiota is more susceptible to environmental influences, such as pollution, lifestyle habits, and antibiotic usage, which can lead to temporary dysbiosis and potentially affect long-term health (Ronan et al., 2021; Hrncir, 2022). The adult gut microbiota tends to be more diverse and stable, but with aging, especially in middle and old age, the diversity of the gut microbiota declines. In older adults, beneficial bacteria decrease while harmful bacteria associated with chronic diseases increase. This shift is closely related to immune system aging and metabolic disorders (Coman and Vodnar, 2020; Sasso et al., 2023). In older adults, the gut microbiota undergoes significant decline. Microbial diversity decreases, and certain harmful bacterial populations may increase, which could contribute to various age-associated diseases. Researcher emphasized that older adults experience dysbiosis due to immune system aging, which leads to chronic inflammation and metabolic diseases (Haran and McCormick, 2021; Malik et al., 2023). The composition of the gut microbiota changes significantly across different life stages, influenced by multiple factors such as immune function, metabolic health, diet, and antibiotic use (Haran and McCormick, 2021). The microbiota is particularly important during infancy for immune development, in childhood for immune and metabolic health, in adulthood for disease prevention, and in older age where its decline is associated with various diseases (Sarkar et al., 2021; Yao et al., 2021).

The abundance of *Bifidobacterium longum subsp. infantis* (BI) and *Bifidobacterium adolescentis* (BA) varies significantly across different life stages (Kato et al., 2017; Salazar et al., 2023; Zhao et al., 2023). BI dominates the gut microbiota in infancy, while BA becomes more abundant as individuals transition to childhood, adulthood, and beyond (Derrien et al., 2019; Chichlowski et al., 2020). Recent research has highlighted their applications and health benefits, focusing on their roles in gastrointestinal health, immune modulation, and potential therapeutic interventions (Hitch et al., 2022; Yaqub et al., 2025). However, comparative studies on the impact of *Bifidobacterium* strains from different age groups on gut microbial diversity and the intestinal immune system, particularly with regard to γδ T cells, are currently insufficient. Our present study employs DSS-induced colitis models, germ-free mouse models, and *in vitro* γδ T cell stimulation assays to comprehensively evaluate the functions of these two strains. We observed significant differences in the impact of *Bifidobacterium* strains from different age groups on the immune system and gut microbiota diversity. The efficacy of microbiota transplantation is influenced by host immune status and microbiota diversity characteristics. Both BI and BA directly interact with γδT cells, with BI inducing the expression of 4-1BB and 4-1BBL on γδT cells. Moreover, among the top 20 significantly altered signaling pathways, both BI and BA are involved in gut inflammation-related pathways, but their effects differ. Our findings provide a theoretical basis for the development of age-associated probiotic intervention strategies, offering new insights into personalized microbiota modulation to enhance immune health and gut homeostasis across different life stages.

## Materials and methods

### Ethical approvals

The human samples were collected from the General Hospital of Ningxia Medical College, and all donors provided a written informed consent before experiments performed. Our present study protocol was conducted in accordance with the Declaration of Helsinki and approved by the Ethics Committee of General Hospital of Ningxia Medical College (approved number: 2020-638). All animal experimental protocols in this study were approved by the Institutional Animal Care and Use Committee of Shanghai Veterinary Research Institute (IACUC approval number: SV-20220/14-02) and were performed in accordance with the ARRIVE guidelines and the Guide for the Care and Use of Laboratory Animals (8th edition, 2011).

### Animals

Female BALB/c mice (6-8 weeks old) were obtained from SPF Biotechnology Co., LTD (Beijing, China). Mice were randomly grouped and each 6 mice were housed in one cage in SPF conditions under a 12h light/12h dark cycle with free access to food and water.

For experimental colitis mouse model, mice received four 7-day cycles of 3% DSS in drinking water, each followed by three 14-day cycles of normal drinking water, according to a previous study with some modifications (Chassaing et al., 2014).

For *Bifidobacterium* intervention experiments, after 1 week of adaptive feeding, mice in the *Bifidobacterium* group received an oral gavage with 2×10^8^ CFU/200 μL *Bifidobacterium* for two consecutive weeks, while mice in the control group were received saline buffer. Two *Bifidobacterium* including *Bifidobacterium longum subsp. infantis* (SHBCC D11206 AS1.1853) *and Bifidobacterium adolescentis* (SHBCC D11107) were selected for the present study, and both of them were purchased from ZHONGKE-JIAYI (Shandong, China) and maintained in our laboratory.

For antibiotic treatment experiments, antibiotic cocktail was prepared to eliminate the initial gut microbiota according to the previous study (Wei et al., 2024). Mice received an oral gavage with 1 mg/kg amphotericin-B for three consecutive days, and then each mouse was given an antibiotic cocktail of 100 mg/kg neomycin trisulfate, 50 mg/kg vancomycin, 100 mg/kg metronidazole and 1 mg/kg amphotericin-B for seven consecutive days. Ampicillin was dissolved into drinking water at a concentration 1 g/L for mice. All antibiotics were purchased from Macklin Biochemical Technology (Shanghai, China) and Solarbio Life Science (Beijing, China). After antibiotic treatment, the mice received *Bifidobacterium* intervention according the methods mentioned above.

After treatments, the mice in each group were euthanized (intraperitoneal injection of 200 mg/kg sodium pentobarbital), and the fence, the colorectal tissue and blood were collected from each group for further experiments.

### γδ T cell isolation and *Bifidobacterium* intervention

Six endoscopic biopsies were obtained from the colons of adult donors undergoing diagnostic colonoscopy. The paracancerous tissues were collected and transported in ice-cold tissue storage solution before cells isolation within two hours. Human γδ T cells were isolated from colonic tissues according to the previous reports (Wu et al., 2017; Rodin et al., 2024), with some modifications. Briefly, tissues were washed using GD medium (STEMERY, China) complemented with 20% fetal bovine serum (FBS) (Gibco, USA), 1% penicillin–streptomycin (Gibco, USA), and cut into small fragments in a sterile dish. The lamina propria lymphocytes were isolated in a collagenase-DNase solution (Sigma, USA) at 37°C for 2 h, and then the cell suspension was then filtered through 70 μm cell strainer twice. After filtration, the cells were cultured in GD medium with 1000 IU/mL IL-2. The cell count, viability and purity were determined using flow cytometry (FCM). For *Bifidobacterium* intervention study, the γδ T cells were exposed to each species of living *Bifidobacterium* for 12 h. Then, the cells were collected for further experiments.

### Enzyme-linked immunosorbent assay

BA and BI were co-cultured with γδ T cells, respectively. The supernatants from different treated groups were collected and centrifuged at 1000×g for 15 minutes to remove particles. The supernatant collected after centrifugation was then used for subsequent experiments. The quantitative assessment of cytokines, including IL-17 and IFN-γ in the cell supernatants was performed using ELISA kits purchased from Abcam (Cambridge, MA), following the protocol provided by the supplier.

### RNA-seq library preparation and sequencing

Total RNA was isolated from different groups using TRIzol (Life Technologies, USA) according to the manufacturer’s instructions. The cDNA libraries were constructed using the NEBNext Ultra II RNA Library Prep Kit (New England Biolabs Inc, USA). Sequencing was performed on the NovaSeq platform. The raw data was processed and filtered using fastq (0.22.0) software, and the clean reads were mapped to the reference genomes using HISAT2 software (http://ccb.jhu.edu/software/hisat2/index.shtml) (Callahan et al., 2016). Differential expression analysis was performed with the DESeq2 (v1.38.3). Transcripts with a |log2Fold Change| > 1 and *p* value < 0.05 were considered significantly differentially expressed mRNA. KEGG and GO analyses for differentially expressed genes were performed using ClusterProfiler (v4.6.0).

### Quantitative real-time PCR validation

To validate the genes differently expressed in different groups, qRT-PCR was used. Briefly, total RNA was extracted from tissue or cell samples using TRIzol (Life Technologies, USA). RNA purity and concentration were determined using a NanoDrop spectrophotometer. qRT- PCR was performed using SYBR^®^ Green Quantitative RT-qPCR mix (Vazyme, China) on a real-time PCR system (ABI QuantStudio5, USA). Gene-specific primers were obtained from Primer Bank (Spandidos et al., 2010) and synthesized commercially by Shanghai Huajin Biotechnology Co., Ltd. The relative expression levels of target genes were calculated using the 2^–ΔΔCt^ method, with β-actin used as the internal control. Each reaction was run in triplicate. The primers used are listed in **Supplementary Table S1**.

### DNA extraction and 16S rRNA gene sequencing

DNA was extracted from fecal samples and colorectal tissue using TIANamp Stool DNA Kit (TIANGEN, Shanghai, China) and VAHTS Universal Pro DNA Library Prep Kit (Vazyme Biotech Co., Ltd., Nanjing, China). The bacteria 16S rRNA gene (variable region V3-V4 region) were amplified with the 16S primers (341F/806R). The PCR products were further purified with VAHTSTM DNA Clean Beads (Vazyme Biotech Co., Ltd., China), and sequenced on an Illumina platform according to the manufacturer’s instructions. The raw data were processed, filtered, denoised, merged, and chimera filtered using the DADA2 plugin (Callahan et al., 2016). Microbiome bioinformatics were analyzed using R package and QIIME2 2019.4 with slight modification (Bolyen et al., 2019).

### Flow cytometric analysis

The levels of CD3+TCRγδ+ T cells, CD3+TCRγ2+ T cells, CD44+TCRγδ+ T cells, CD25+TCRγδ+ T cells, CD3+CD25+ T cells and CD3+CD44+ T cells were detected by flow cytometry (FCM). The samples were prepared as previously reported (Lu et al., 2022; Rodin et al., 2024). Briefly, the mice were euthanized, the blood and the colon tissue were collected. Blood was lysed using a lysis solution (TIANGEN, China), and the tissues were ground using the plunger end of the syringe. After three washes, the cell suspensions were then incubated with their respective primary antibodies (anti-CD3-FITC, anti-CD25-PE, anti-TCRγδ-BV421, anti-TCRγ2-APC, anti-CD44-PerCP-cy5.5) for 30 min in the dark at 4°C, followed by three washes with staining buffer. Finally, the cells were resuspended in 200 μL of staining buffer for flow cytometry analysis (Beckman CytoFLEX, USA). The data were analyzed with FlowJo software (Tree Star, USA). All the antibodies were obtained from BD Biosciences (Becton Dickinson, USA).

### Hematoxylin and eosin and immunofluorescence staining

H&E staining was used to analyze microanatomy, and IF were used to analyze protein expression of tissues and organs (Hinton et al., 2019; Zaqout et al., 2020). Briefly, the colon tissues from different groups were collected and immediately fixed with 4% paraformaldehyde. After fixation, samples were dehydrated through a graded ethanol series, cleared in xylene, and embedded in paraffin. Tissue sections were fixed and stained with hematoxylin to visualize nuclei, followed by eosin to stain the cytoplasm. Slides were imaged using Nikon NIS-Elements microscopy.

Three immunofluorescent markers main principle is based on Tyramide signal amplification (TSA). Briefly, tissue sections were permeabilized with 0.1% Triton X-100 and blocked with 5% bovine serum albumin (BSA). Primary antibodies (anti-TGFβ1, anti-IL10, anti-FOXP3) were applied and incubated overnight at 4°C. After washing, sections were incubated with fluorophore-conjugated secondary antibodies for 1 hour at room temperature. Nuclei were counterstained with DAPI, and images were captured using Nikon microscope (NIKON ECLIPSE C1).

### Statistical analysis

GraphPad Prism 8 (GraphPad Software, USA) and Origin 2019b software (OriginLab, MA, USA) were used to generate common chart and data statistics. Data are presented as mean ± SD. The statistical differences between groups were analyzed using Student’s *t* test or one-way ANOVA. A *p* value < 0.05 was considered statistically significant.

## Results

### Immune homeostasis significantly changed in DSS-induced chronic colitis

**Figure 1:**
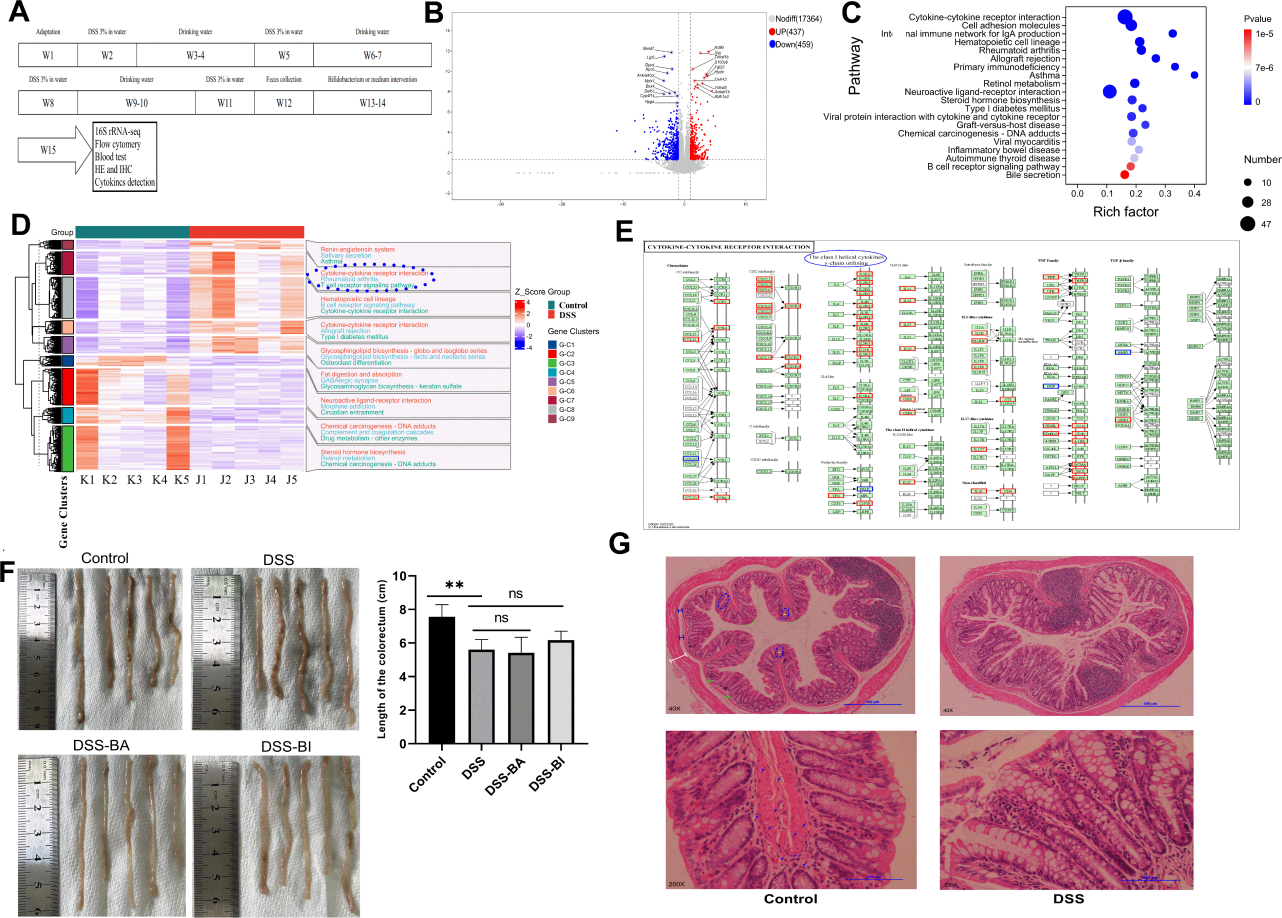
The transcriptional characteristics of DSS-induced colitis in mice. **(A)** A flowchart illustrating the construction of a chronic colitis mouse model and the intervention with *Bifidobacterium*. **(B)** Volcano plots comparing the Control group and the DSS group. **(C)** KEGG enrichment scatter plot of differentially expressed genes (DEGs). The color of the dots indicates the p-value, while the size of the dots represents the number of enriched genes. **(D)** Cluster analysis heatmap of DEGs and sample groups. Genes are displayed horizontally, and each column represents an individual sample. The intensity of red reflects higher gene expression levels, while blue indicates lower expression levels. Expression levels were normalized using zscore calculations. **(E)** Genes enriched in the cytokine-cytokine receptor interaction pathway. Upregulated genes are marked in red, and downregulated genes are marked in blue. DEGs associated with the g chain are highlighted with a blue circle. **(F)** Colorectal length measurements in the different groups. **(G)** Representative images of H&E stained colon sections from control and DSS groups. The top row is the original patches, and the bottom row is corresponded images produced after augmentation. The blue circle denotes the intestinal crypt, while the green dotted line outlines the crypt surface. The submucosal layer is shown in blue, with the crypt base represented by a green “U.” The white line indicates the muscle layer, the yellow short line marks the crypt width, and the blue arrow highlights immune cells. **p < 0.01.

DSS-induced chronic colitis is a mouse model for studying intestinal inflammation, commonly used to investigate clinically relevant intestinal inflammation, such as ulcerative colitis (UC) and inflammatory bowel disease (IBD). To verify the effects of *Bifidobacterium* from different age on induced colitis in mice, *Bifidobacterium longum subsp. infantis* (BI) and *Bifidobacterium adolescentis* (BA) were selected, and DSS-induced chronic colitis model was established in BALB/c mice (**Figure 1A**), and interventions were conducted using both types of *Bifidobacterium*. Transcriptome sequencing was performed on colonic tissues isolated from different groups, and 16S sequencing was conducted on gut microbiota. The comparison between the DSS group (J1-J5) and the PBS group (K1-K5) revealed 896 differentially expressed genes (DEGs), comprising 437 upregulated and 459 downregulated genes, as shown in **Figure 1B**. The top 20 enriched KEGG pathways included cytokine-cytokine receptor interaction and inflammatory bowel disease signaling pathways (**Figure 1C**). KOBAS and KEGG were used to annotate pathway heatmaps, cytokine-cytokine receptor interaction signaling pathways were annotated (**Figure 1D**). Analysis of DEGs within the KEGG cytokine pathway revealed significant upregulation of γ-chain-related cytokines (**Figure 1E**). The γ-chain is a core component of the γδ T cell receptor (TCR), determining antigen recognition patterns and functional characteristics of γδ T cells along with the δ-chain (Li et al., 2021; Xin et al., 2024). The γ-chain not only serves as the structural core of γδ T cells but also confers unique immune functions through its diversity, signaling, and metabolic regulation properties.

Furthermore, the length of the colon was measured. Compared to the control group, the DSS-induced colitis model showed a significant shortening of the colon length (**Figure 1F**). The result of H&E staining found that DSS exposure damages intestinal crypts, corrupts intestinal barrier integrity and increases immune cell infiltration (**Figure 1G**).

In conclusion, DSS intervention in the gut significantly alters the intestinal immune status, resulting in a reduction in colorectal length. DSS induced aberrant expression of cytokines, indicating changes in specific cell subpopulations.

### *Bifidobacterium* intervention significantly reduced the level of γδ T cell infiltration in the colorectum

**Figure 2:**
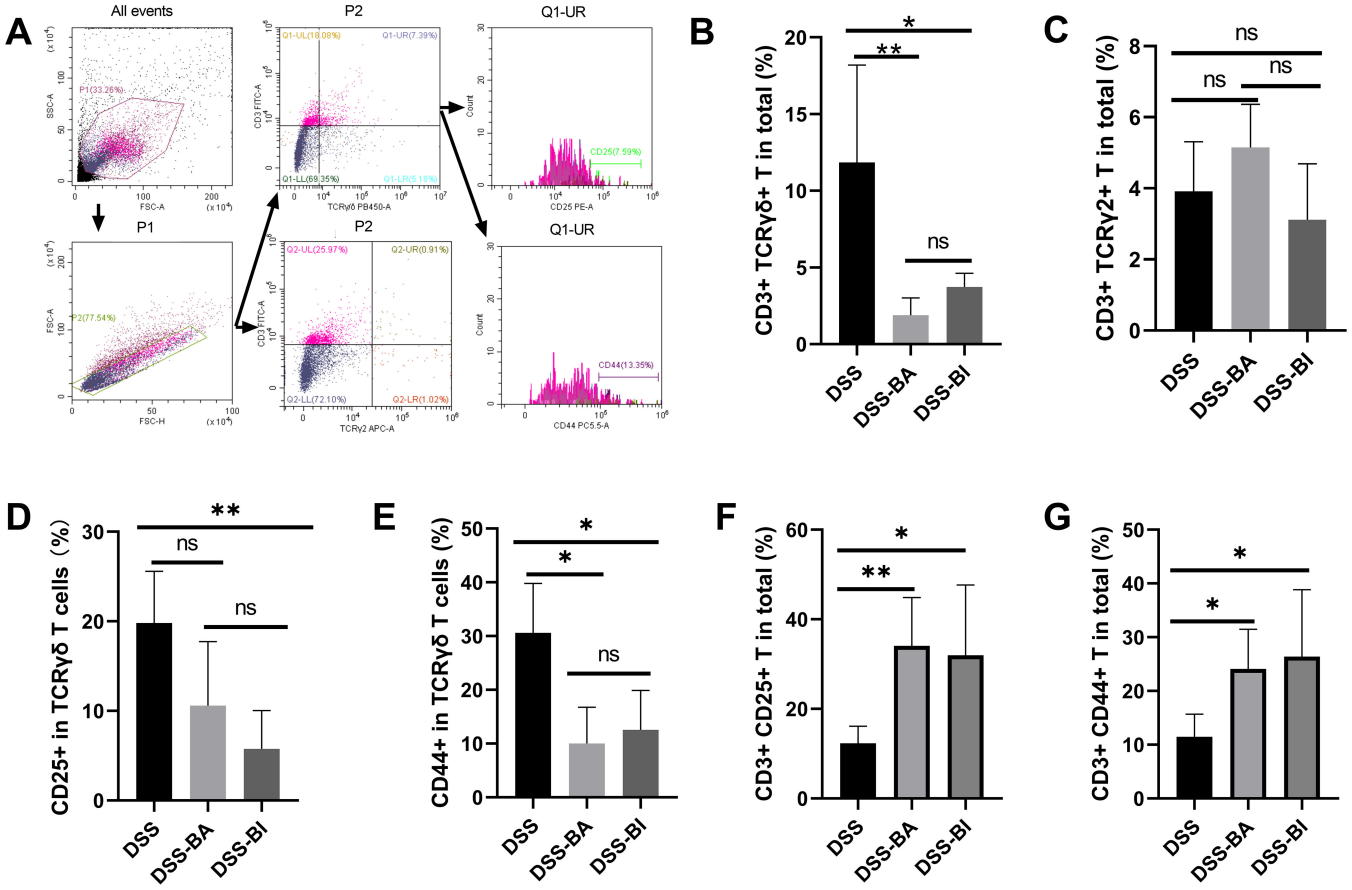
*Bifidobacterium* interventions influenced the γδ T cell levels in the colorectum in a DSS-induced mouse model. **(A)** Gating strategy for flow cytometry analysis of the colorectum. **(B)** Percentage of γδ T cells in the colorectum across different groups. **(C)** Percentage of TCR γ2+ T cells in the colorectum across different groups. **(D)** Percentage of CD25+ cells within γδ T cells in the colorectum. **(E)** Percentage of CD44+ cells within γδ T cells in the colorectum. **(F)** Percentage of CD3+ CD25+ cells in the colorectum. **(G)** Percentage of CD3+ CD44+ cells in the colorectum. ns, not significant, *p < 0.05, **p < 0.01.

The γδ T cells play a crucial role in the innate immune system, especially in mucosal immunity, where they are involved in immune responses in the gut (Kang et al., 2023). To determine whether T cell responses to BI and BA isolated from different stages of life, flow cytometry was used to analyze the level and functions of γδ T cells in peripheral blood and colorectal tissue after BA and BI interventions in colitis models (**Figure 2A**; **Supplementary Figure S1**). Our results showed that BA and BI reduced the infiltration of γδ T cells in the gut (**Figure 2B**), but had no significant impact on the levels of the TCR γ2+ γδ T cell subset (**Figure 2C**). Further analysis revealed that BA and BI interventions decreased the expression of CD25 and CD44 in γδ T cells (**Figures 2D, E**). However, when examining CD3+ T cells overall, we found that the expression levels of CD25 and CD44 were significantly elevated (**Figures 2F, H**), suggesting the involvement of other immune cell subsets, possibly through immune suppression by regulatory T cells (Tregs).

### BA and BI effectively alleviate DSS-induced abnormalities in peripheral blood cell subsets and mitigate pathological damage in intestinal tissues

**Figure 3:**
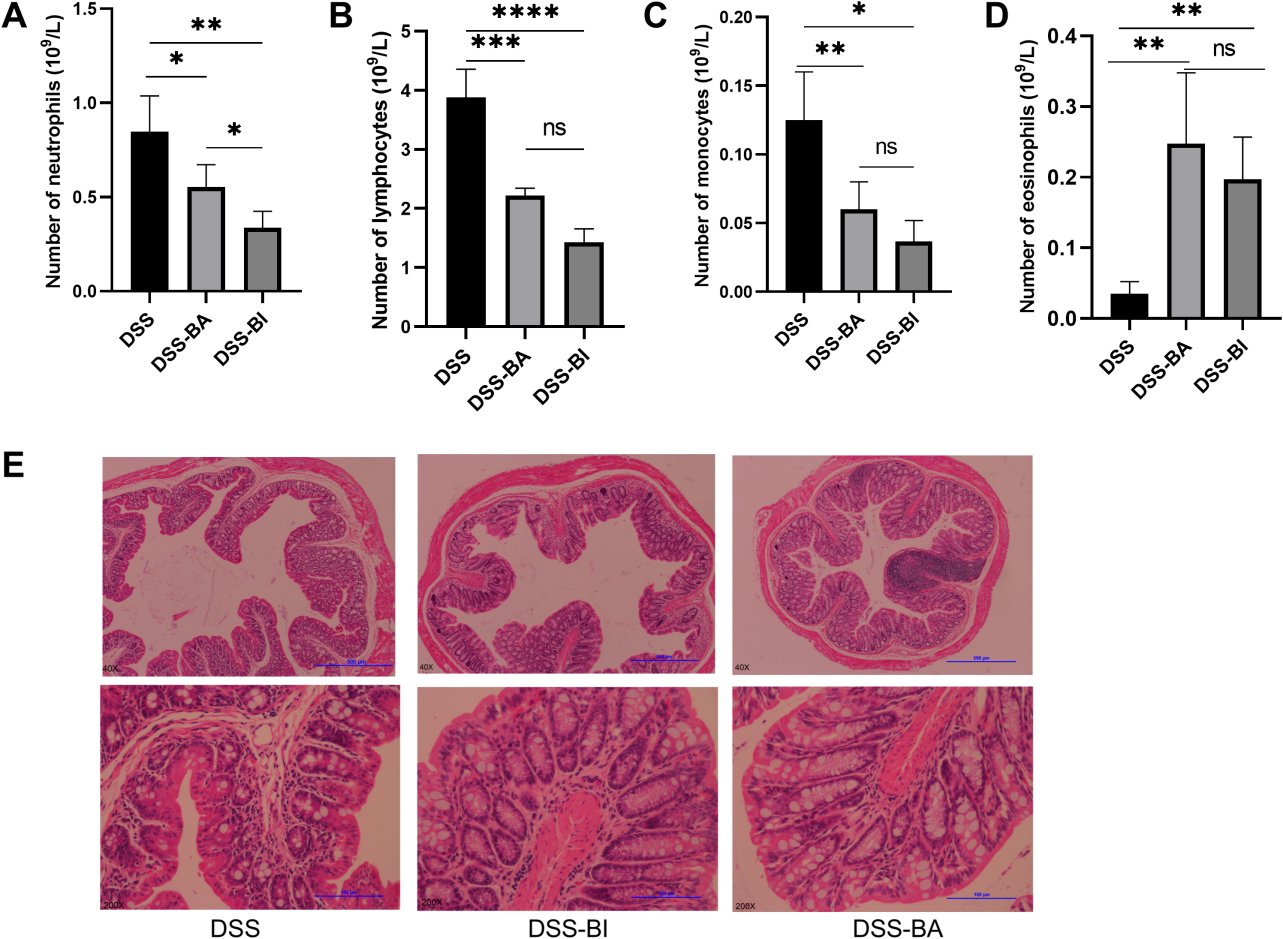
**(A)** Neutrophil count, **(B)** lymphocyte count, **(C)** monocyte count, and **(D)** eosinophil count was analyzed using the Mindray BC-5300 auto hematology analyzer in peripheral blood mononuclear cells (PBMCs). **(E)** Representative images of H&E stained colon sections from DSS, DSS-BA and DSS-BI groups. The top row is the original patches, and the bottom row is corresponded images produced after augmentation. *p < 0.05, **p < 0.01, ***p < 0.001, ****p < 0.0001, ns, not significant.

Hematological profiles are often used as reference indicators for disease diagnosis and as factors for predicting prognosis. The total numbers of neutrophil cells, lymphocyte cells, monocyte cells, and eosinophil cells were analyzed using the Mindray BC-5300 auto hematology analyzer. Results showed that the number of neutrophil cells, lymphocyte cells, and monocyte cells decreased following both BA and BI intervention (**Figures 3A–C**), while the number of eosinophil cells increased (**Figure 3D**). Compared to control group, DSS treatment significantly shortened the colorectal length (**Figure 1F**). However, short-term (2 weeks) interventions with BA or BI did not affect colorectal length (**Figure 1F**), suggesting that changes in colorectal length may require a prolonged repair process. H&E staining results showed that both BA and BI treatments promote the recovery of damaged intestinal crypts, enhance intestinal barrier integrity, and decrease immune cell infiltration (**Figure 3E**). These findings underscore the potential of BI and BA in alleviating intestinal inflammation and promoting the repair of intestinal mucosal barrier integrity and crypts. However, the restoration of colorectal length may necessitate a more prolonged healing process.

### Oral administration of BI and BA triggered different immune response in a DSS-induced colitis mouse model

**Figure 4:**
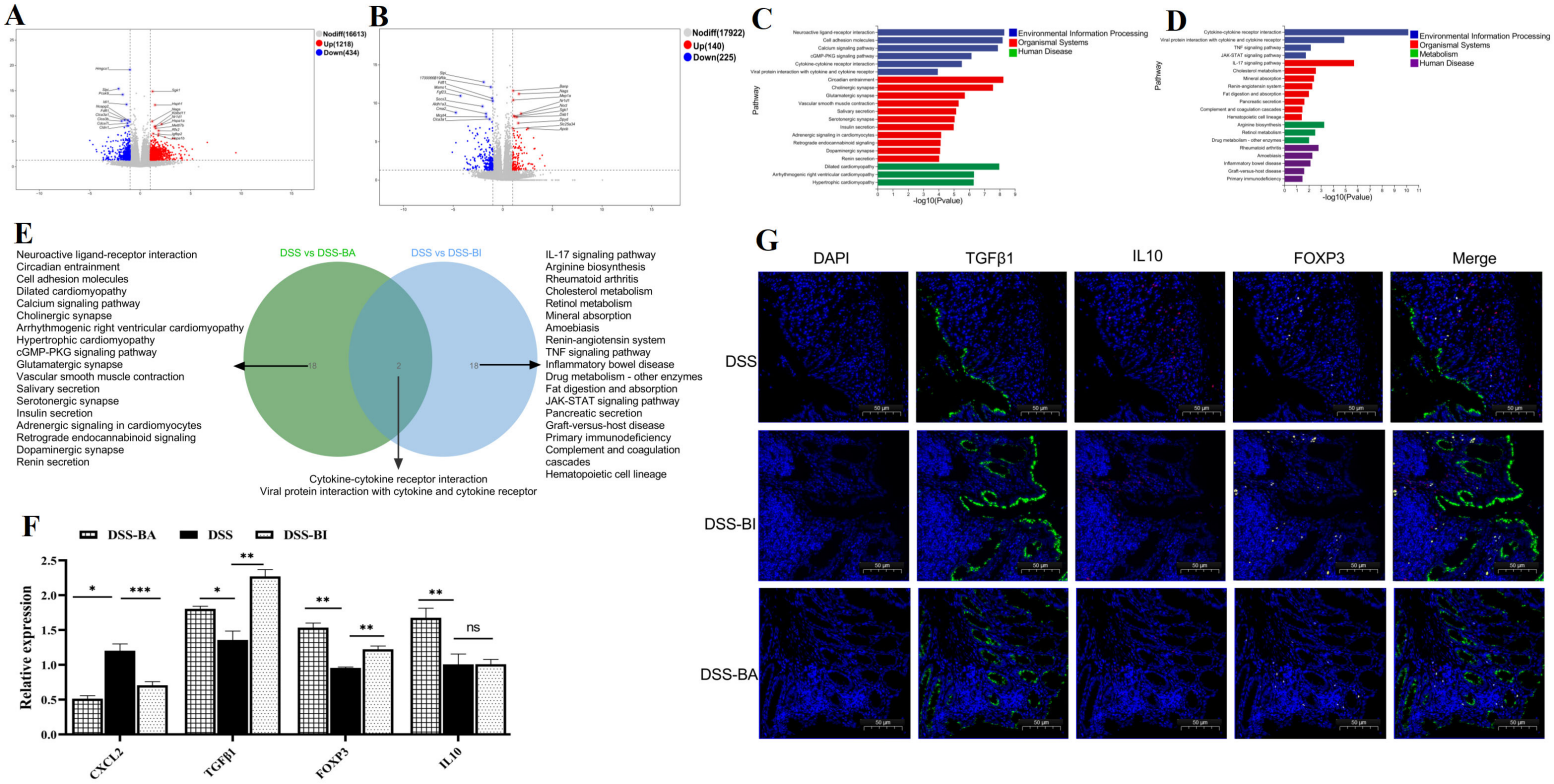
*Bifidobacterium* intervention altered the transcriptional response in the colorectum in a DSS-induced mouse model. Volcano plots comparing the DSS-BA vs DSS groups **(A)**, and DSS-BI vs DSS groups **(B)**. Genes with significant upregulation are highlighted in red, whereas those with significant downregulation are shown in blue. Top 20 KEGG enrichment bar chart of DEGs from DSS-BA vs DSS groups **(C)**, and DSS-BI vs DSS groups **(D)**. The vertical axis represents KEGG pathways, and the horizontal axis defaults to KEGG enrichment (-log10 p-value). **(E)** Venn diagram illustrating the overlap between DSS vs BA and DSS vs BI groups. **(F)** Further validation of the RNA seq using qRT-PCR. * *p*<0.05, ** *p*<0.01, ***p < 0.001, ns, not significant. **(G)** Immunofluorescence staining analysis of immune-related genes in tissues isolated from DSS, DS-BA and DSS-BI groups.

To explore gene expression patterns and identify significantly altered genes following BA and BI intervention, we performed RNA-seq of the colon tissue samples obtained from PBS (DSS group), *Bifidobacterium longum subsp. infantis* (DSS-BI group), and *Bifidobacterium adolescentis* (DSS-BA group) treated in a DSS-induced colitis mice model. Volcano plots revealed that 1218 upregulated and 434 downregulated DEGs were identified between DSS-BA and DSS group (**Figure 4A**). Between the DSS-BI and DSS groups, 365 DEGs were identified, including 140 up-regulated and 225 down-regulated genes (**Figure 4B**). KEGG pathway enrichment analysis was performed to elucidate the associated signal pathways of DEGs, and the top 20 signaling pathways were illustrated in **Figures 4C, D**. Results showed that there was a great significant difference between DSS-BA and DSS groups, in particular, the signaling pathway in DSS-BA group was highly significant in Neuroactive ligand-receptor interaction, Cytokine-cytokine receptor interaction, Calcium signaling pathway and Cell adhesion molecules (**Figure 4C**). In the comparison between DSS-BI and DSS groups, the most enriched KEGG pathways were Cytokine-cytokine receptor interaction, IL-17 signaling pathway, TNF signaling pathway, Inflammatory bowel disease and so on (**Figure 4D**). Additionally, clustering analysis was performed based on the expression levels of the DEGs, and further delineated the expression patterns of the DEGs across the DSS-BA, DSS-BI and DSS groups (**Supplementary Tables S2**, **S3**).

A Venn diagram revealed that a total of 38 signaling pathways were significantly enriched in the comparisons of DSS-BA vs. DSS and DSS-BI vs. DSS (**Figure 4E**). Among these, two signaling pathways, including Cytokine-cytokine receptor interaction and Viral protein interaction with cytokine and cytokine receptor, were shared between two comparisons. BA and BI primarily modulated intestinal immunity through distinct signaling pathways. Even within the same pathway, the differential gene expression profiles induced by each strain exhibited notable differences. These findings suggested that BA and BI may influence the regulation of immune cell subsets and their functions via distinct mechanisms. To further validate the expression changes of immune-related genes identified through transcriptomic analysis, four DEGs were randomly selected using qRT-PCR. The results demonstrated that CXCL2, a major chemokine involved in colitis progression, showed a significant reduction following BA and BI interventions in a DSS induced mouse model. Three genes closely related to intestinal inflammation, including TGFβ1, FOXP3, and IL10 showed significant increase following BA and BI interventions (**Figure 4F**). The validation of qRT-PCR was consistent with the RNA-seq data.

Furthermore, the protein expression of these three DEGs associated with intestinal inflammation was assessed using immunofluorescence. Results confirmed the upregulation of TGFβ1 and FOXP3 following BA and BI treatments in the DSS-induced model, with TGFβ1 exhibiting a shift in expression to the intestinal crypt region (**Figure 4G**).

### *Bifidobacterium* altered the dynamic changes in gut microbiota

To investigate the effect of BA and BI on gut microbiota in DSS-induced colitis mouse model, we analyzed the gut microbiome difference among Control, DSS, DSS-BA and DSS-BI groups using 16s rRNA seq. Sequencing results showed that as the number of samples increased, the number of ASVs tended to plateau, which confirmed the sufficiency of sampling and the viability of subsequent data analysis (**Supplementary Figure S2A**). The PCoA showed a distinct segregation of microbial communities into four separate clusters among the different groups, reflecting indicating significant differences in the composition of microbiota within each group (**Supplementary Figure S2B**). In **Supplementary Figure S2C**, the number of common ASVs sequences shared among the four groups was 157.

To determine the specific taxonomic biomarkers in different groups, we further performed LEfSe analysis. Results revealed distinct microbial signatures, with notable genera exhibiting significance across different groups (**Supplementary Figure S2D**). Specifically, at the genus, *Eubacterium_F* and *Phocea*, UBA11471, *Aerococcus* and *Ileibacterium* emerged as significant genera characterized in DSS-BA group, while *Nanosyncoccus*, *Lactococcus_A*, *Vagococcus_B*, *Ruthenibacterium*, *Pseudogracilibacillus*, *Gemella* and *Paenalcaligenes* exhibited significance within the DSS-BI group (**Supplementary Figure S2D**). Taxonomic tree in packed circles illustrating the overall abundance of taxa at different levels, revealed distinct differences in the abundance and distribution of taxa among different groups. *Bacilli*, *Clostridia* and *Bacteroidia* were the main bacterial community in all the groups (**Supplementary Figure S2E**). Furthermore, we compared the composition of different groups. At the genus level, *Alistipes_A* and *Lactobacillus* were found a boost in the DSS group, compared with the Control group, but *Ligilactobacillus* and *CAG-485* showed a contrary trend (**Supplementary Figure S2F**). In additionally, we discovered that *Lactobacillus* showed a significant decrease in DSS-BA group, compared to the DSS group, while there was no significant difference after the intervention of BI (DSS-BI vs DSS group) (**Supplementary Figure S2F**).

These data demonstrated that both BA and BI interventions modulated gut microbiota composition in DSS-induced colitis model, but they did so in distinct ways. The unique enrichment of specific genera in the DSS-BA and DSS-BI groups suggested that BA and BI may exert strain-specific effects on the gut microbial ecosystem. The decrease in *Lactobacillus* following BA intervention, contrasted with the stable levels post-BI intervention, indicated that these probiotics may differentially influence certain bacterial populations.

### *Bifidobacterium* isolated from different age groups exhibited distinct transcriptomic profiles following intervention in germ-free mice model

**Figure 5:**
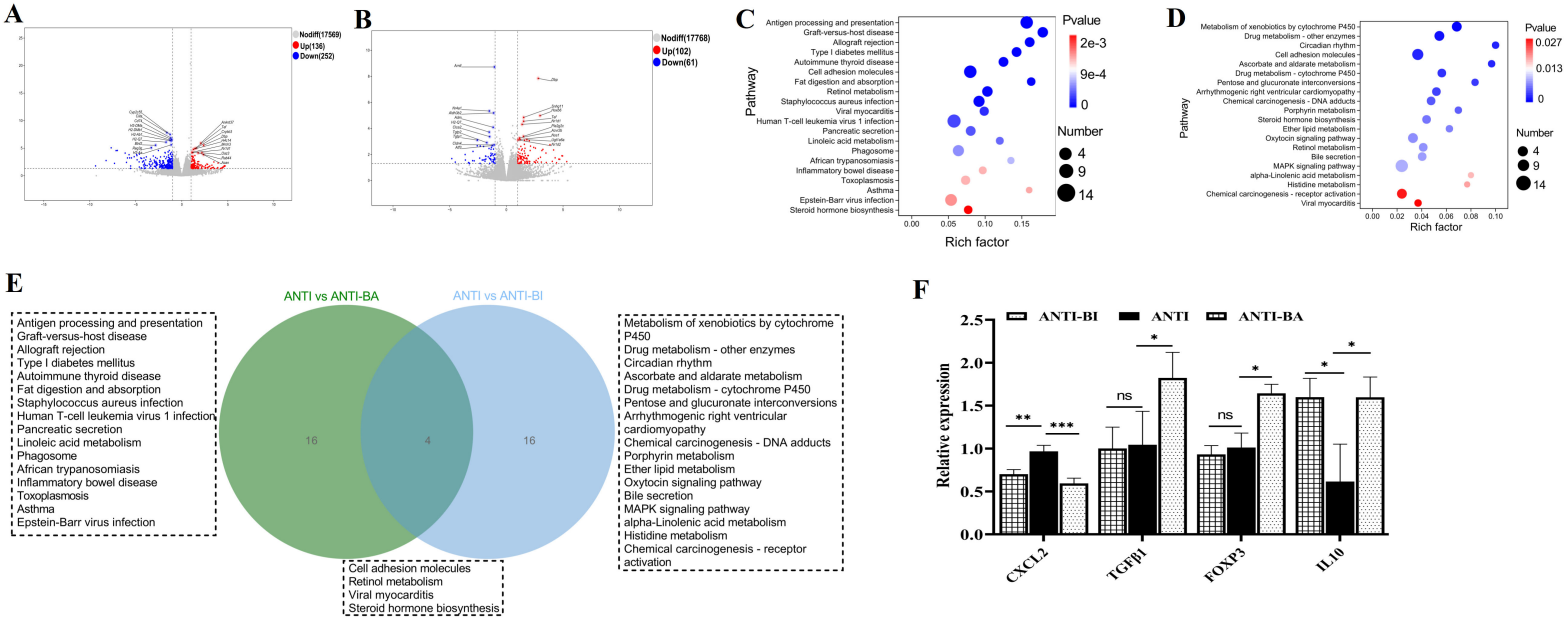
Gene expression analysis after BA and BI intervention in an antibiotic-treated germ-free mouse model. Volcano plots of identify DEGs following BA **(A)** and BI intervention **(B)**. Top 20 KEGG enrichment plots from ANTI-BA vs ANTI groups **(C)**, and ANTI-BI vs ANTI groups **(D)**. **(E)** Venn diagram illustrating the overlap of the top 20 enriched pathways between the BA and BI interventions. **(F)** Further validation of the RNA seq using qRT-PCR. *p < 0.05, **p < 0.01, ns, not significant.

*Bifidobacterium* contributes to both intestinal immune regulation and the regulation of intestinal microbiota (Sun et al., 2020; Gavzy et al., 2023). To investigate the specific effects of *Bifidobacterium* on intestinal health, we established a germ-free mouse model by depleting gut microbiota with antibiotic cocktail. Germ-free mice were then used to evaluate the intervention effects of BA (ANTI-BA group) and BI (ANTI-BI group), with an antibiotic-treated group serving as the control (ANTI group). Volcano plots revealed that 388 and 163 DEGs were identified in the ANTI-BA vs Anti group (**Figure 5A**), and ANTI-BI vs ANTI group (**Figure 5B**), respectively. KEGG clusters analysis showed that these DEGs between ANTI-BA and ANTI groups were mainly enriched in Antigen processing and presentation, Cell adhesion molecules, Retinol metabolism, Linoleic acid metabolism and Phagosome (**Figure 5C**), while those DEGs between ANTI-BI and ANTI groups were mainly enriched Metabolism of xenobiotics by cytochrome P450, Cell adhesion molecules, MAPK signaling pathway and Chemical carcinogenesis-receptor activation (**Figure 5D**). Furthermore, the clustering analysis showed a significant difference in the transcripts between the ANTI-BA and ANTI groups (**Supplementary Table S4**), as well as between ANTI-BI and ANTI groups (**Supplementary Table S5**). Additionally, the overlapping enrichment pathways in the ANTI-BA vs ANTI and ANTI-BI vs ANTI groups were visualized by a Veen diagram. Results showed that 4 overlapping pathways were obtained, including Cell adhesion molecules, Retinol metabolism, Viral myocarditis and Steroid hormone biosynthesis (**Figure 5E**). These results demonstrated that BA and BI exerted distinct influences on host gene expression and immune pathways, reflecting their unique probiotic properties. Furthermore, four DEGs were selected for further validation by qRT-PCR, and the results were consistent with the RNA-seq data (**Figure 5F**).

### *Bifidobacterium* isolated from different age groups modulated the gut microbiota in germ-free mice model

To minimize interference from other gut microorganisms, we established an antibiotic-cleared germ-free mouse model. Subsequently, we assessed the effects of BA and BI interventions on the gut microbiota using 16S rRNA sequencing. Rarefaction analysis showed that the ASVs in each group approached saturation as the number of samples increased (**Supplementary Figure S3A**). As shown in **Supplementary Figure S3**, we found a higher bacterial diversity was observed in the gut of Control group than that in ANTI, ANTI-BA and ANTI-BI groups (Chao 1 index). The PCoA based on the weighted_unifrac revealed the dissimilarity of microbial communities among the samples from different groups (**Supplementary Figure S3B**). Especially, the dissimilarity of microbial communities was obvious after antibiotic and *Bifidobacterium* treated groups (ANTI, ANTI-BA, ANTI-BI groups), compared the control group. A Venn diagram revealed that 389 ASVs were shared among all four groups, while 5131, 3290, 1926 and 2439 ASVs were sole to control, ANTI, ANTI-BA, ANTI-BI group, separately (**Supplementary Figure S3C**). To investigate the featured gut bacterial microbes that differed different groups, we performed random forest analysis, which is an ensemble classifier based on a machine-learning algorithm. Results showed that *Enterococcus_B* genus was the most important taxa among the top 15 genera. Among these, seven genera including *Adlercreutzia*, *Bifidobacterium*, QWKK01, *Paludicola*, *Romboutsia_B*, *Limivicinus*, *Soleaferrea* were mainly enriched in ANTI-BI group, while *Enterococcus_B* and *Mammaliicoccus* were more abundant in ANTI-BA group (**Supplementary Figure S3D**). Furthermore, the composition in different groups were analyzed. At the TOP 10 genera, the gut microbiota in control group was dominated by *Ligilactobacillus* (6.84%), *Duncaniella* (7.12%), *Amulumruptor* (7.97%), CAG-485 (7.89%). However, the most abundant genera in the ANTI groups were *Lactobacillus* (11.11%) and *Dubosiella* (9.79%). *Lactobacillus* and *Dubosiella* were significantly increased in ANTI group, compared to the control group (**Supplementary Figure S3E**). Compared to ANTI group, these two genera maintain higher levels after BA intervention, while *Dubosiella* was significantly reduced after BI intervention. In conclusion, both BA and BI significantly influence microbial diversity in germ-free mouse models. Notably, short-term interventions with either BA or BI do not fully restore the microbial composition and diversity to that of the control group.

### *Bifidobacterium* directly regulated γδ T cells function

**Figure 6:**
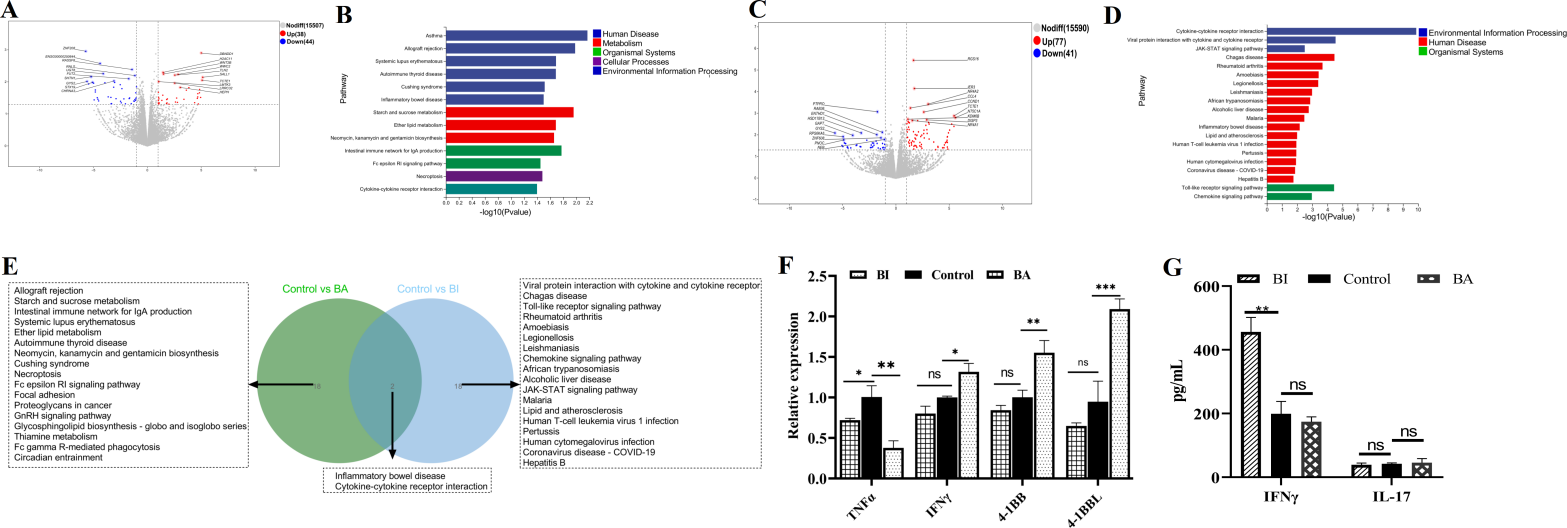
Transcriptome analysis of γδ T cells following treatment with BA and BI *in vivo*. Volcano Plots: These plots depict the DEGs in the BA **(A)** and BI **(B)** groups, compared to the control group, with significantly upregulated genes highlighted in red and downregulated genes in blue. Top 20 KEGG pathway enrichment analysis showing the enriched KEGG pathways following BA **(C)** and BI interventions **(D)**. The vertical axis represents the KEGG pathways and the horizontal axis indicates the -log10(p-value) for pathway enrichment. **(E)** Venn diagram displays the overlap between the gene sets identified from the comparisons (BA vs C groups) and (BI vs C groups). **(F)** Further validation of the RNA seq using qRT-PCR. **(G)** Concentrations of IFN-γ and IL-17 across various treatment groups. ns, not significant. *p < 0.05, **p < 0.01, ***p < 0.001, ns, not significant.

Beyond antigen-presenting cell-dependent activation by bacteria and their metabolites, mucosal damage and increased intestinal permeability due to inflammation or tumors enable direct contact between *Bifidobacteria* and γδ T cells. To exclude/minimize the influence of the intestinal immune microenvironment, *Bifidobacteria* with intestinal γδ T cells co-culture model was established to investigate the activation effects. In the comparative transcriptomic analysis, a total of 82 DEGs, including 38 upregulated and 44 downregulated genes, were identified in the control group (C group) and *B. adolescentis* treated group (BA group) (**Figure 6A**). The DEGs were then subjected to KEGG enrichment analysis to understand the alteration of signaling pathways in the γδT cells treated with *B. adolescentis*. The KEGG pathway enrichment analysis identified 136 important pathways, and significantly enriched pathways were mainly associated with the Cytokine-cytokine receptor interaction, Necroptosis, Cushing syndrome and Fc epsilon RI signaling pathway (**Figure 6B**). Clustering analysis further confirmed a significant difference in the transcripts and signaling pathways between the BA and C groups (**Supplementary Table S6**). As shown in **Figure 6C**, a total of 118 DEGs were identified between the C group and *B. infantis* treated group (BI group), with 77 upregulated genes and 41 downregulated genes in the BI group compared to the C group. To further investigate the key signaling pathways, we performed KEGG enrichment analysis. The top 20 KEGG pathway were found to be enriched in three KEGG A classes: Organismal systems, Human disease and Environmental information processing (**Figure 6D**). The significantly enriched pathways were primarily associated with Cytokine-cytokine receptor interaction, Viral protein interaction with cytokine and cytokine receptor, JAK-STAT signaling pathway, Toll-like receptor signaling pathway and Chemokine signaling pathway (**Figure 6D**). Clustering analysis confirmed a significant difference in the transcripts and signaling pathways between the BI and C groups (**Supplementary Table S7**). Subsequently, Venn diagram revealed that 2 KEGG pathways, including Inflammatory bowel disease and Cytokine-cytokine receptor interaction, were overlapped between BA vs C and BI vs C groups, while 18 pathways were sole to BA vs C and BI vs C groups, respectively (**Figure 6E**). Furthermore, four immune-related DEGs were random selected for qRT-PCR validation. Results showed that BI treatment led to a significant increase in the expression of 4-1BB, 4-1BBL, and IFN-γ, while significantly reducing TNFα expression. In contrast, BA stimulation did not significantly affect 4-1BB, 4-1BBL, or IFN-γlevels, though it did reduce TNFα expression (**Figure 6F**). These results were also consistent with the RNA-seq data. Furthermore, BA and BI were co-cultured with γδ T cells, and IFN-γ and IL-17 cytokine secretion levels in the supernatant were measured using the human IL-17 or IFN-γ ELISA kit. The results showed that BI directly stimulated γδ T cells to increase IFN-γ secretion, but did not affect IL-17 expression. In contrast, BA had no significant effect on the secretion of either cytokine when directly stimulating γδ T cells (**Figure 6G**). Previous studies have reported that BA can influence the expression of IFN-γ and IL-17, suggesting that *Bifidobacterium* may regulate the expression of these cytokines either by affecting other immune cells or indirectly modulating γδ T cell function through antigen-presenting cells (APCs) (Gavzy et al., 2023; Li et al., 2025). According to the results, both BA and BI directly induced changes in T-cell immune-related signaling pathways, suggesting that *Bifidobacterium* or its metabolites can directly participate in the regulation of γδ T cells without relying on other immune cell subsets. However, the effects of BA and BI on γδ T cells differ, among the top 20 enriched KEGG signaling pathways, only two are shared between both interventions.

### The regulatory effects of BA and BI on associated signaling pathways were dependent on the immune microenvironment

**Figure 7:**
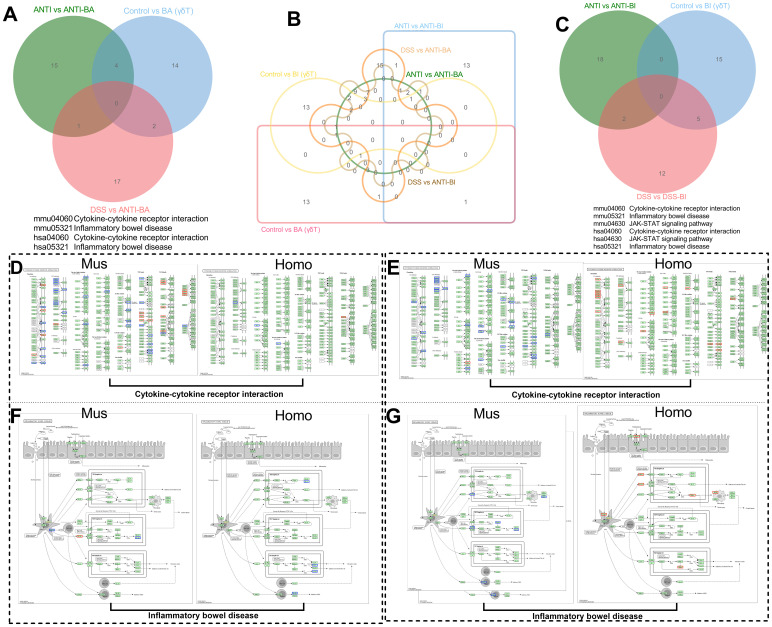
Combined analysis of enriched signaling pathways and corresponding genes following interventions with BA and BI across three models. Venn diagrams illustrating the top 20 KEGG pathways enriched with differentially expressed genes in the two mouse models following BA **(A)** and BI **(C)** intervention. **(B)** Venn diagram depicting the top 20 KEGG pathways enriched with DEGs common to all three models after BA and BI intervention. Schematic representation of DEGs enriched in the Cytokine-Cytokine Receptor Interaction pathway following BA **(D)** and BI **(E)** intervention. Mouse data are shown on the left, human data on the right; blue indicates down-regulated expression, and red indicates up-regulated expression. Schematic representation of DEGs enriched in the Inflammatory Bowel Disease pathway following BA **(F)** and BI **(G)** intervention. Mouse data are shown on the left, human data on the right; blue indicates down-regulated expression, and red indicates up-regulated expression.

To further compare and validate the immunoregulatory roles of BA and BI, we conducted a combined analysis of the enriched signaling pathways across three models’ post-intervention: the DSS-induced colitis mouse model, the germ-free mouse model, and the *in vitro* human intestinal γδ T cell co-culture model. Venn diagrams were constructed using the top 20 significantly altered KEGG pathways from each model to identify common pathways shared among two or all three models. Specifically, we generated Venn diagrams illustrating: KEGG pathways post-BA intervention across the three models (**Figure 7A**), all differential KEGG pathways post-BA and post-BI interventions across the three models (**Figure 7B**), and KEGG pathways post-BI intervention across the three models (**Figure 7C**).

The selection criterion for common pathways involved identifying the top 20 significantly altered pathways in the *in vitro* human intestinal γδ T cell co-culture model that overlapped with those in at least one of the other two models. This approach yielded four BA-associated pathways and six BI-associated pathways. Among the shared pathways, including Cytokine-cytokine receptor interaction and Inflammatory bowel disease-the corresponding differentially expressed genes were mapped onto both murine and human KEGG pathways (**Figures 7D–G**). These results revealed significant differences in the impact of BA and BI on the same pathway, both *in vivo* and *in vitro*.

Notably, BA predominantly activated the NOD2-like receptor signaling pathway both *in vivo* and *in vitro*, with upregulation of the NOD2 gene (**Figure 7F**). In the DSS-induced colitis model, BI also activated the NOD2-like receptor signaling pathway, leading to increased NOD2 expression. In the *in vitro* co-culture model, BI simultaneously activated both NOD-like receptor (NLR) and Toll-like receptor (TLR) signaling pathways (**Figure 7G**). The *in vitro* co-culture model demonstrated that BA had a minimal effect on γδ T cells, suggesting that γδ T cells may not be the direct targets of BA or its metabolites. Conversely, co-culture with BI directly activated γδ T cells (**Figure 7G**), and significantly enhancing the expression levels of 4-1BB and 4-1BBL (**Figure 6F**), which may provide insights for generating highly active γδ T cells *in vitro*. In the DSS-induced colitis *in vivo* model, BI primarily activated immunosuppressive signaling pathways.

### The interactions between intestinal microbes and top 20 KEGG signal pathways immune related genes

To further explore the interactions between host and gut microbiome, we conducted a correlation analysis between DEGs associated with immune regulation and microbial taxa at the genus level by using a multi-omics correlation analysis (**Supplementary Figure S4**). As shown in **Supplementary Figure S4A**, we observed significant correlations (*p* < 0.05) between the majority of DEGs and the relative abundance of *Ileibacterium*, *Erysipelatoclostridium*, Borkfalk*ia*, UBA11471, CAG_873, CAG_83, *Acetatifactor*, *Ruthenibacterium* and *Stoquefichus* (DSS-BA vs DSS group). The majority of DEGs (DSS-BI vs DSS group) involved in the immune regulation showed a positive correlation with the relative abundance of *Merdisoma*, CAG_83, *Acetatifactor*, *Pseudobutyricicoccus* and *Peptococcus*. Additionally, only several notable negative correlations were observed between these DEGs and dominant taxa, including *Ruminococcus_B*, *Nanosyncoccus*, *Harryflintia* (**Supplementary Figure S4B**). In the germ-free mouse model, we found that the main microbiota showing positive correlations with immune response-related DEGs (ANTI-BA vs ANTI group) included *Acetitomaculum*, *Limenecus*, *Ruthenibacterium*, *Sporofaciens*. Only three DEGs- H2-BI, H2-DMa and Cldn9 exhibited negative correlations with *Bifidobacterium*, *Enterococcus_B*, QWKK01 and *Ruthenibacterium* (**Supplementary Figure S4C**). Moreover, for ANTI-BI vs ANTI groups, we picked up all 17 DEGs enriched in immune-related pathways and highlighted their correlation to 28 significantly differential genera. The results showed that the expression of 17 DEGs was significantly correlated with the relative abundances of at least one genus. Notably, five DEGs (Fos, Cldn4, Gadd45g, Nr4a1, Cd274) were only correlated with two genera (*Limenecus* and *Ructibacterium*) of 28 differential genera (**Supplementary Figure S4D**). In our multi-omics correlation analysis, we identified significant associations between DEGs related to immune regulation and various gut microbial genera. These findings suggested that specific gut microbial genera may play distinct roles in modulating immune responses under different experimental conditions.

## Discussion

Research indicates that the composition and diversity of the gut microbiota exhibit distinct age-associated characteristics. Notably, the abundance of *Bifidobacterium* varies significantly across different age groups. *Bifidobacteria*, a family of bacteria isolated from both plants and animals, display unique distribution patterns at various life stages (Grosicki et al., 2018; Bradley and Haran, 2024). While numerous studies have explored the effects of probiotics or prebiotics, such as *Bifidobacteria*, on gut microbiota (Hidalgo-Cantabrana et al., 2017; Wang et al., 2020), there is a lack of systematic research on how dominant *Bifidobacteria* strains enriched in from different age groups influence gut immunity and microbial diversity.

Our present study employed three models, including the DSS-induced colitis model, germ-free mouse model, and the *in vitro* human intestinal γδ T cell co-culture model, to systematically assess the effects of *Bifidobacteria* derived from different age groups. We investigated changes in gut microbial composition and abundance, intestinal transcriptomic features, and alterations in relevant immune cell subsets and their functions following *Bifidobacterial* intervention. Our results revealed significant differences in how *Bifidobacteria* from various age groups, specifically strains BA and BI, rebuild gut microbiota ecosystem and activate immune-related signaling pathways, both within the same model and across different models. In both *in vivo* models, short-term continuous probiotic interventions did not restore gut microbial levels and diversity to normal. In the DSS-induced colitis model, DSS treatment led to a shortening of the colorectal length, and short-term interventions with either *Bifidobacteria* strain failed to return it to normal levels. Transcriptomic data from the DSS-induced model identified γδ T cells as key responders, with significant activation of γ-chain utilizing genes.

Although DSS is the primary agent for inducing colitis, it does not directly affect bacteria (Krause et al., 2024), suggesting that DSS primarily influences the gut microbial environment indirectly by affecting the intestinal immune milieu. Analysis of transcriptomic data indicated that *Bifidobacteria* from different age groups differentially impacted gut immunity and microbial diversity. In-depth studies of probiotics from various age groups could facilitate more precise treatments for intestinal inflammations, such as IBD across different age demographics. The effects of single or multiple probiotic strains on intestinal microbial diversity and immunity may depend on the specific intestinal environment of the host from which they were originally isolated. When using mixed probiotic preparations, it is essential to consider the competition and coordination between microorganisms. Additionally, microbial interventions should account for potential incompatibilities, similar to drug interactions.

The role of TLR and NLR signaling pathways in food allergies, chronic intestinal diseases and autoimmune conditions has been studied (Hitch et al., 2022; Kawai et al., 2024). However, there is a lack of research on the mechanisms through which *Bifidobacteria* and their metabolites regulate these signaling pathways in immune cells. Both strains modulated key signaling pathways, including those involved in cytokine-cytokine receptor interactions and inflammatory bowel disease. Notably, while BA predominantly activated the NOD2-like receptor signaling pathway, BI not only activated the NOD2-like receptor signaling pathway in the DSS-induced colitis model but also simultaneously triggered additional NLR and TLR signaling pathways in the *in vitro* co-culture model.

The *in vitro* co-culture data provided further insights into strain-specific effects on γδ T cells. BA exhibited minimal direct effects on γδ T cells, suggesting that its immunomodulatory role might be mediated indirectly, potentially through other immune cells or via modifications of the local cytokine milieu. In contrast, BI directly activated γδ T cells, as evidenced by significant increases in the expression of activation markers 4-1BB and 4-1BBL. This direct activation indicates that BI or its metabolites could serve as a potential therapeutic tool to generate highly active γδ T cells *in vitro*, which might be harnessed for novel immunotherapies.

Previous studies have shown that *B. infantis* can reduce enteric inflammation of premature infants (Underwood et al., 2014; Henrick et al., 2020; Nguyen et al., 2021). While *B. adolescentis* primarily supports gut health in adults, although it has also been found to effectively mitigate the occurrence of inflammatory bowel disease (IBD) in premature neonatal rats by regulating immune responses (Wu et al., 2017). Our results indicated that both BA and BI effectively mitigate intestinal tissue pathological damage in DSS-induced colitis mouse model. Notably, BI demonstrated a more robust activation of γδ T cells in the cell culture model. These findings suggest that BA and BI may play distinct roles in inflammatory processes. However, our study is limited by the absence of age-enriched bacterial strains, particularly those from elderly individuals, for comparative analysis. Given that both gut microbiota and γδ T cells exhibit age-related variations, future research should focus on clinically correlating specific metabolites from age-matched *Bifidobacterium* strains with the functional characteristics and levels of γδ T cells derived from individuals of the same age group. This approach could deepen our understanding of the reciprocal regulation between gut microbiota and *Bifidobacterium*, ultimately facilitating the development of targeted microbial therapeutics and more effective treatments for related diseases. Another limitation of our study is the lack of metabolomics data, which constrains mechanistic interpretation. Previous studies have shown that microbial metabolites, particularly phosphoantigens (pAgs) such as hydroxy-methyl-butyl-pyrophosphate (HMBPP) and isopentenyl pyrophosphate (IPP), can activate γδ T cells and modulate immune responses (Henrick et al., 2020; Nguyen et al., 2021; Hitch et al., 2022; Kawai et al., 2024). *Escherichia coli*, for example, produces specific pAgs that stimulate γδ T cells (Underwood et al., 2014). Upon pAg stimulation, γδ T cells upregulate HLA-DR expression and exert various immune functions (Henrick et al., 2020). Therefore, future studies should focus on exploring both the direct and indirect effects of metabolic products from different *Bifidobacterium* species on γδ T cell–mediated immune regulation.

Overall our findings underscore the pivotal role of the immune microenvironment in shaping responses to probiotic interventions and reveal distinct mechanistic pathways by which different *Bifidobacterium* strains modulate immune responses.

## Conclusions

Our present study examined the immunomodulatory effects of *Bifidobacterium adolescentis* (BA) and *Bifidobacterium longum subsp. infantis* (BI) on gut immunity and microbial diversity across three models: DSS-induced chronic colitis, germ-free mice, and *in vitro* human intestinal γδ T cell co-culture. Transcriptomic analysis revealed strain-specific modulation of cytokine signaling pathways, with BA activating NOD2-like receptor signaling and BI enhancing IL-17 and TNF pathways. Multi-omics analysis further indicated that both probiotics influenced immune responses through microenvironment-dependent mechanisms, providing potential therapeutic insights for inflammatory gut diseases.

## Data Availability

The sequencing data have been deposited in the Sequence Read Archive (SRA), under the asscession number: PRJNA12492430 (https://www.ncbi.nlm.nih.gov/bioproject/PRJNA12492430). The original contributions presented in the study are included in the article/**Supplementary Material**. Further inquiries can be directed to the corresponding authors.

## Supplementary material

Supplementary Figure 1 *Bifidobacterium* interventions had a minimal impact on the function of innate γδ T cell subsets in the blood in a DSS-induced mouse model. **(A)** Gating strategy for flow cytometry analysis of the blood. **(B)** Percentage of γδ T cells in the blood across different groups. **(C)** Percentage of TCR γ2+ T cells in the blood across different groups. **(D)** Percentage of CD25+ cells within γδ T cells in the blood. **(E)** Percentage of CD44+ cells within γδ T cells in the blood. **(F)** Percentage of CD3+ CD25+ cells in the blood. **(G)** Percentage of CD3+ CD44+ cells in the colorectum. ns: not significant, **p* < 0.05, ***p* < 0.01.

Supplementary Figure 2 *Bifidobacterium* intervention altered the gut microbiota in a DSS-induced mouse model. **(A)** Rarefaction curve of the estimated number of genera using the Chao1 index. **(B)** Principal coordinates analysis (PCoA) analysis. Each point in the figure represents a sample, and points of different colors indicate different groups. **(C)** Venn diagram of ASVs. Different colors represent different groups. **(D)** The bar graph and cladogram indicate the taxa that discriminate among the groups. Only taxa with an alpha value of 0.05 and with absolute LDA (log10) scores >2.0 were considered significant. **(E)** Taxonomic tree in packed circles. The largest circle represents the phylum level, and the gradually smaller circles represent class, order, family, genus, and species according to the gradient. The larger the sector area, the higher the abundance of the taxon in the corresponding group. **(F)** The ordinate is the relative abundance of the genus and the abscissa is the name of each sample in the grouping scheme.

Supplementary Figure 3 Analysis of gut microbial diversity after BA and BI intervention based on 16S sequencing in a germ-free mouse model. **(A)** Rarefaction curve of the estimated number of genera using the Chao1 method. **(B)** PCoA analysis. Each data point represents an individual sample, with color coding distinguishing between different groups. **(C)** Venn diagram of ASVs. Different colors represent different groups. **(D)** Random Forest plot showing the 15 most predictive bacterial genera that differentiate different groups. **(E)** Relative Abundance in Genus. The y-axis represents the relative abundance of each genus, while the x-axis displays sample names, categorized according to the grouping scheme.

Supplementary Figure 4 Interactions between the gut microbes and DEGs related to immune regulation under different conditions. Correlation heatmap of DEGs and gut microbiota from DSS-BA vs DSS groups **(A)**, DSS-BI vs DSS groups **(B)**, ANTI-BA vs ANTI groups **(C)**, ANTI-BI vs ANTI groups **(D)**. Mouse data are shown on the left, human data on the right; blue indicates down-regulated expression, and red indicates up-regulated expression.
