# Correction: Immune microenvironment-dependent effects of age-associated Bifidobacterium strains on gut immunity and microbial diversity

**DOI:** 10.3389/fcimb.2026.1809832

**Published:** 2026-03-25

**Authors:** Yaqin He, Furui Zhang, Zhiqiang Tian, Ruyi Li, Ming Su, Liping Hong, Jun Wen, Cao Zhang, Jinhai Tian, Le Guo

**Affiliations:** 1 General Hospital of Ningxia Medical University, Yinchuan, Ningxia, China; 2 School of Laboratory Medicine, Ningxia Medical University, Yinchuan, Ningxia, China; 3 Department of Oncology II, General Hospital of Ningxia Medical University, Yinchuan, Ningxia, China; 4 Department of Gastrointestinal Surgery Affiliated Hospital of Ningxia Medical University, Yinchuan, China; 5 Medical Science Research Institute, General Hospital of Ningxia Medical University, Yinchuan, China

**Keywords:** γδ T, *Bifidobacterium longum subsp*., *infantis*, *Bifidobacterium adolescentis*, DSS, gut microbial

There was a mistake in **Figure 1** as published. In the original study design, animals in the Control, DSS, DSS-BA, and DSS-BI groups were treated and analyzed simultaneously as part of the same experimental batch. For improved clarity and manuscript structure, the corresponding results were presented separately in **Figure 1F** and **Figure 3E**. Consequently, the DSS group image appears in two different figures. Importantly, this does not represent image duplication or data reuse, but rather the repeated display of the same experimental condition to support different analytical contexts. No images were reused to represent different samples, no data were repurposed across experimental groups, and no conclusions are affected. To avoid any potential misunderstanding, we kindly request that an explicit explanatory note be added to the manuscript. Similarly, to facilitate readers’ understanding of intestinal morphology, we created an illustrative schematic diagram (**Figures 1G**, schematic diagram) by integrating the image at 40× magnification from the Control group in **Figure 1G** and the image at 200× magnification from the DSS group in **Figure 3F**. Consequently, schematic diagram in **Figure 1G** appears twice. We explicitly clarify that this schematic diagram is not a duplicated experimental image and does not represent raw data. The same schematic illustration appears more than once in the article solely for explanatory purposes. No experimental images were duplicated, and no original data were reused or relabeled. To prevent misinterpretation, we respectfully request that this clarification be added to the manuscript. The corrected **Figure 1** and **Figure 3** appears below.

**Figure 1:**
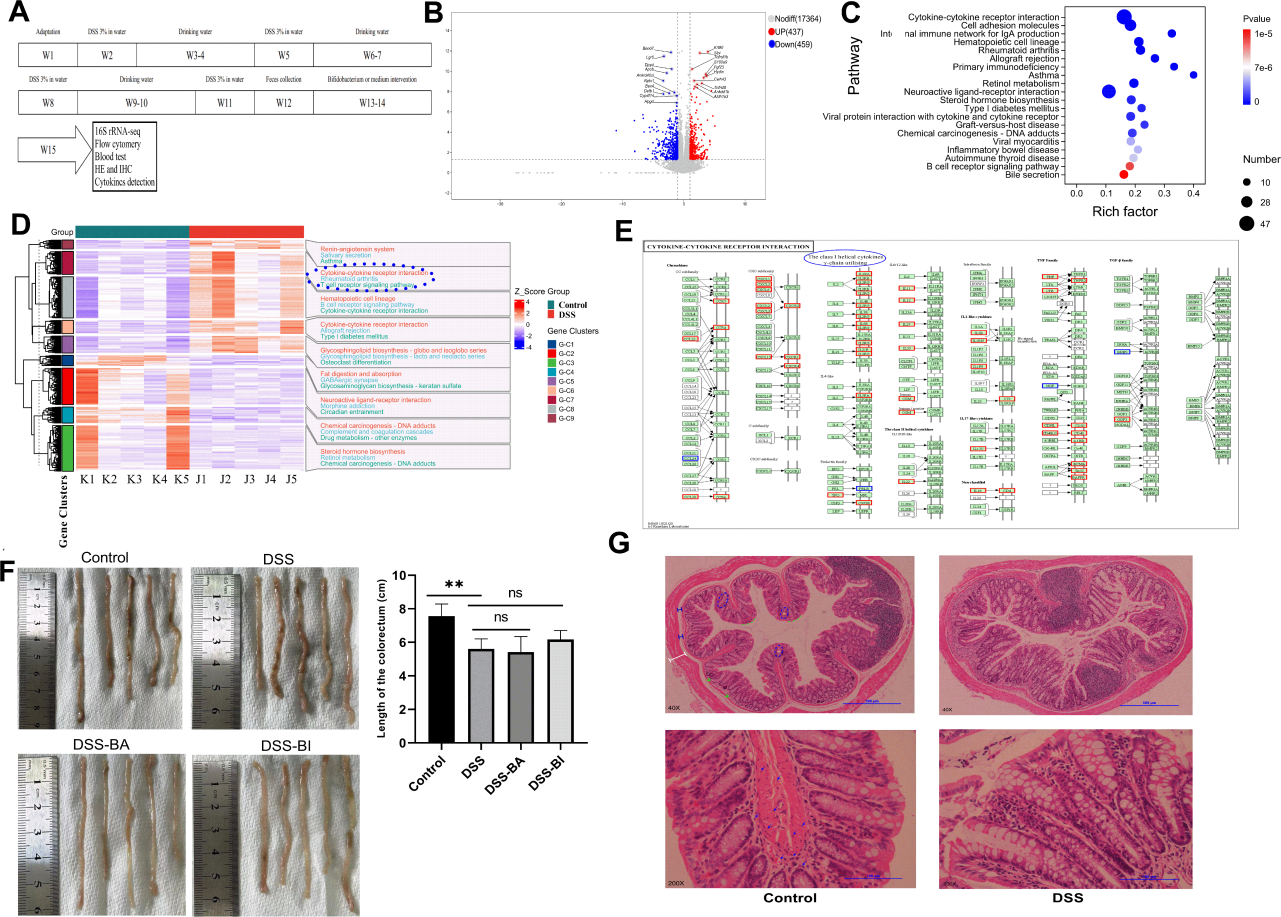
The transcriptional characteristics of DSS-induced colitis in mice. **(A)** A flowchart illustrating the construction of a chronic colitis mouse model and the intervention with *Bifidobacterium*. **(B)** Volcano plots comparing the Control group and the DSS group. **(C)** KEGG enrichment scatter plot of differentially expressed genes (DEGs). The color of the dots indicates the p-value, while the size of the dots represents the number of enriched genes. **(D)** Cluster analysis heatmap of DEGs and sample groups. Genes are displayed horizontally, and each column represents an individual sample. The intensity of red reflects higher gene expression levels, while blue indicates lower expression levels. Expression levels were normalized using z-score calculations. **(E)** Genes enriched in the cytokine-cytokine receptor interaction pathway. Upregulated genes are marked in red, and downregulated genes are marked in blue. DEGs associated with the γ chain are highlighted with a blue circle. **(F)** Colorectal length measurements in the different groups. **(G)** Representative images of H&E stained colon sections from control and DSS groups. The top row is the original patches, and the bottom row is corresponded images produced after augmentation. The blue circle denotes the intestinal crypt, while the green dotted line outlines the crypt surface. The submucosal layer is shown in blue, with the crypt base represented by a green “U.” The white line indicates the muscle layer, the yellow short line marks the crypt width, and the blue arrow highlights immune cells. **p < 0.01.

**Figure 3:**
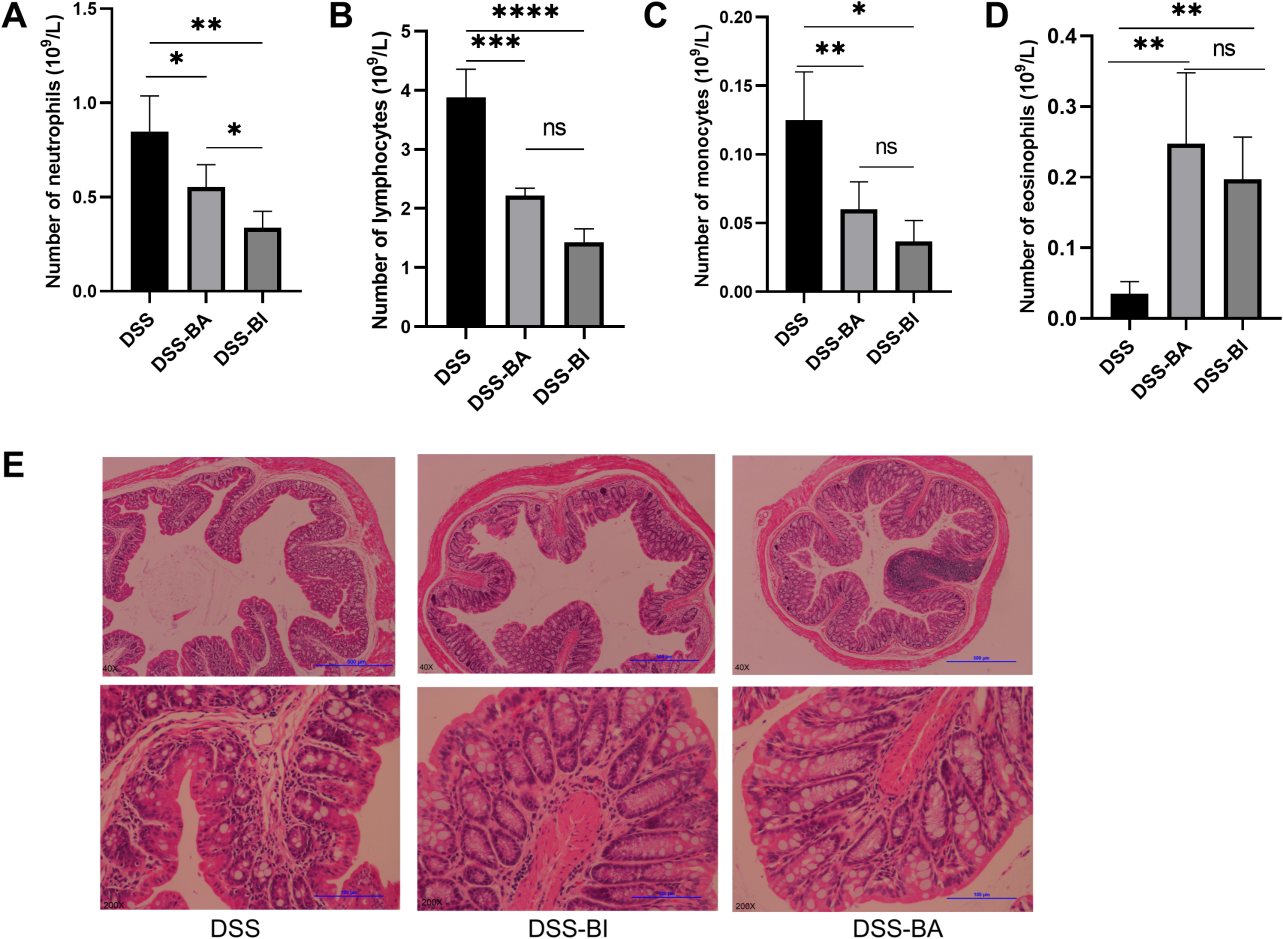
**(A)** Neutrophil count, **(B)** lymphocyte count, **(C)** monocyte count, and **(D)** eosinophil count was analyzed using the Mindray BC-5300 auto hematology analyzer in peripheral blood mononuclear cells (PBMCs). **(E)** Representative images of H&E stained colon sections from DSS, DSS-BA and DSS-BI groups. The top row is the original patches, and the bottom row is corresponded images produced after augmentation. *p < 0.05, **p < 0.01, ***p < 0.001, ****p < 0.0001, ns, not significant.

The original version of this article has been updated.

There was a mistake in the caption of **Figure 1F** as published. “(F) Colorectal length measurements in the Control and DSS groups.” The corrected caption of **Figure 1F** appears below.

“(F) Colorectal length measurements in the different groups”

There was a mistake in the caption of **Figure 3E, F** as published. “(E) Colorectal length measurement in different groups. (F) Representative images of H&E stained colon sections from DSS, DSS-BA and DSS-BI groups.” The corrected caption of **Figure 3E** appears below.

“(E) Representative images of H&E stained colon sections from DSS, DSS-BA and DSS-BI groups”

There was a mistake in the caption of **Figure 3E, F** as published. However, short-term (2 weeks) interventions with BA or BI did not affect colorectal length (**Figure 3E**), suggesting that changes in colorectal length may require a prolonged repair process. H&E staining results showed that both BA and BI treatments promote the recovery of damaged intestinal crypts, enhance intestinal barrier integrity, and decrease immune cell infiltration (**Figure 3F**). The corrected caption of **Figure 3E** appears below.

“However, short-term (2 weeks) interventions with BA or BI did not affect colorectal length (**Figure 1F**), suggesting that changes in colorectal length may require a prolonged repair process. H&E staining results showed that both BA and BI treatments promote the recovery of damaged intestinal crypts, enhance intestinal barrier integrity, and decrease immune cell infiltration (**Figure 3E**)”

The original version of this article has been updated.

