# Supplementary figures and images for: Immune microenvironment-dependent effects of age-associated Bifidobacterium strains on gut immunity and microbial diversity
Source: Front Cell Infect Microbiol. 2025 Sep 16;15:1639178. doi: 10.3389/fcimb.2025.1639178 (PMC12479543; doi:10.3389/fcimb.2025.1639178)

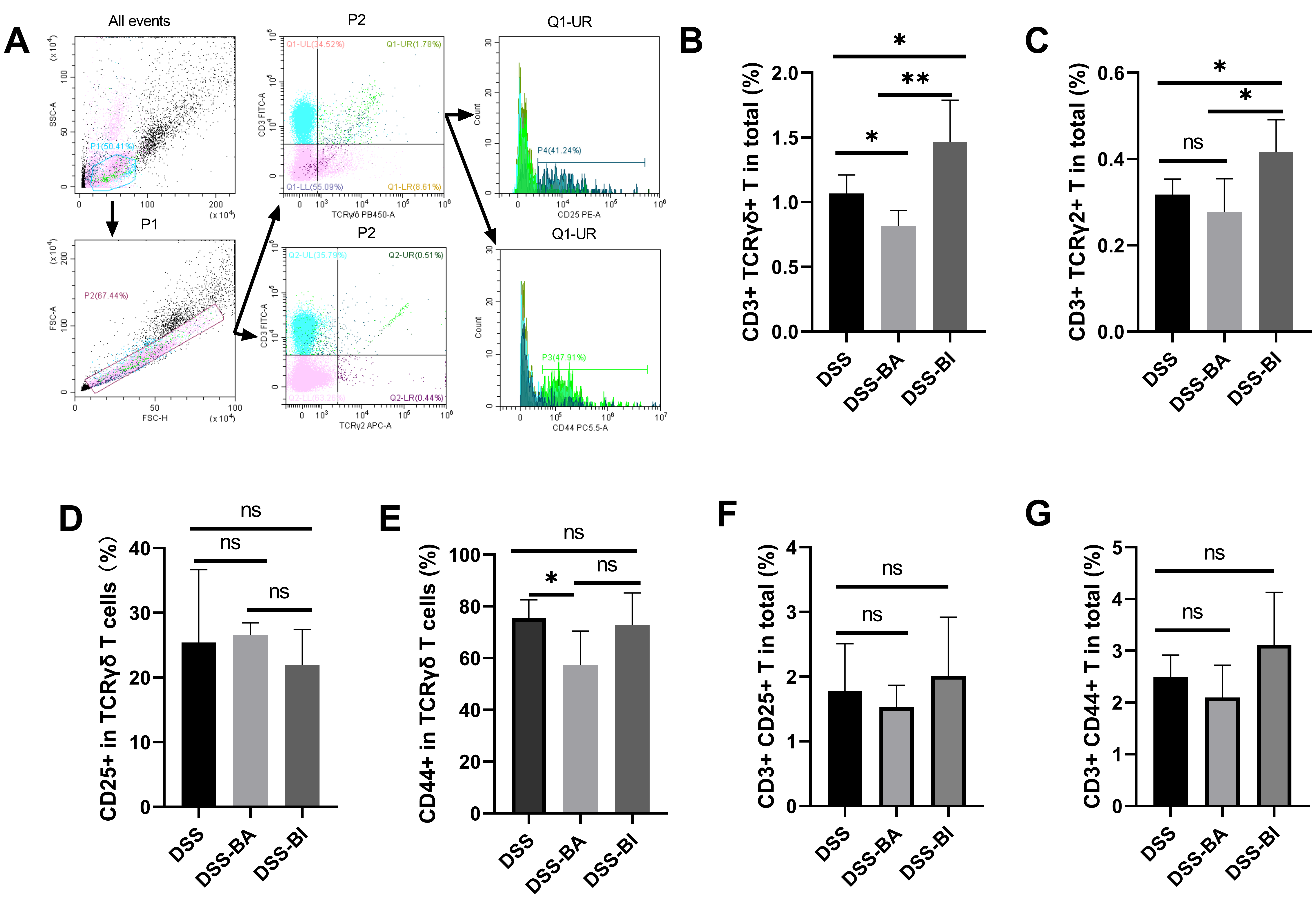

Supplement: Supplementary Figure 1 — Bifidobacterium interventions had a minimal impact on the function of innate γδ T cell subsets in the blood in a DSS-induced mouse model. (A) Gating strategy for flow cytometry analysis of the blood. (B) Percentage of γδ T cells in the blood across different groups. (C) Percentage of TCR γ2+ T cells in the blood across different groups. (D) Percentage of CD25+ cells within γδ T cells in the blood. (E) Percentage of CD44+ cells within γδ T cells in the blood. (F) Percentage of CD3+ CD25+ cells in the blood. (G) Percentage of CD3+ CD44+ cells in the colorectum. ns: not significant, *p < 0.05, **p < 0.01. [file Image1.tif]

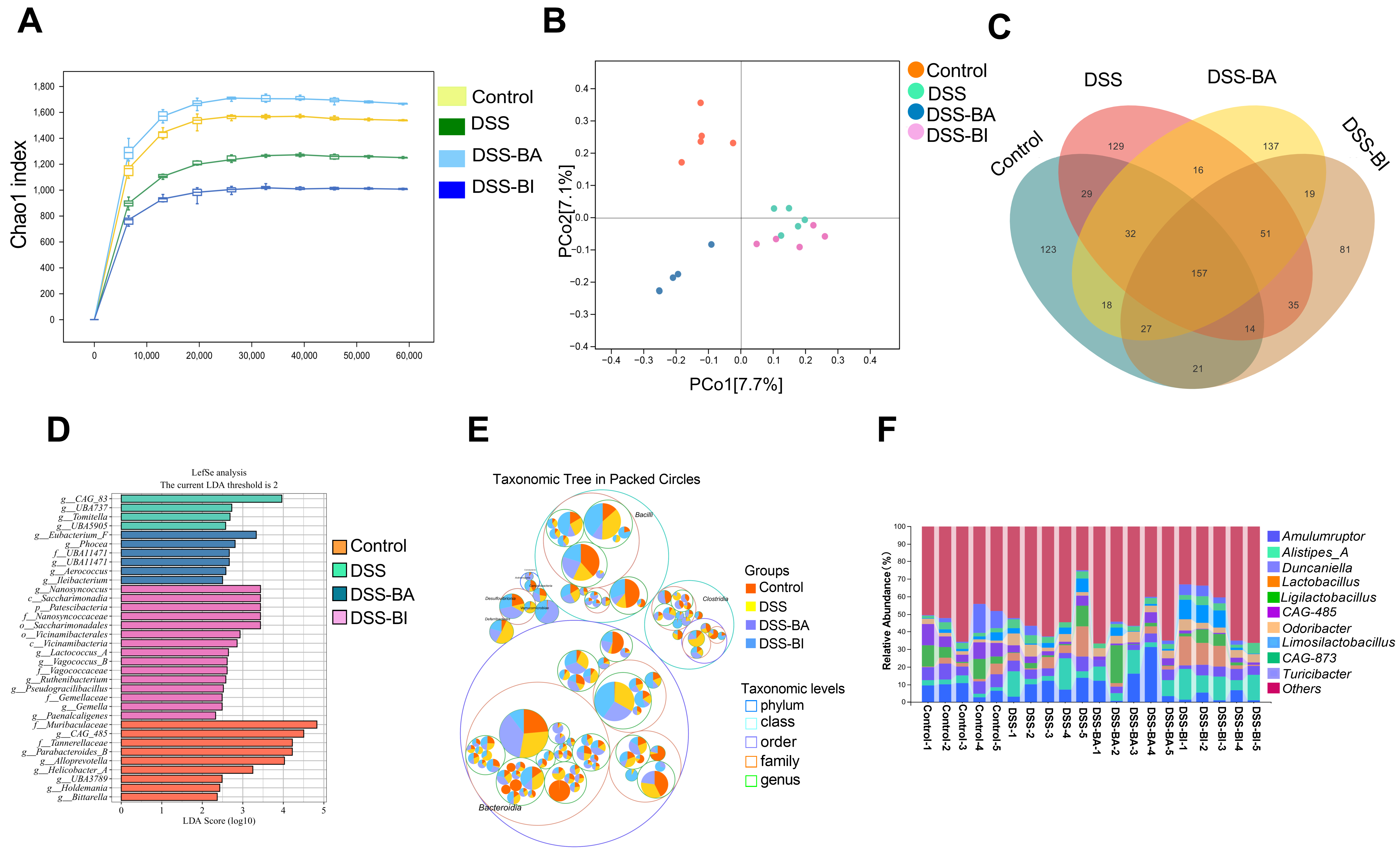

Supplement: Supplementary Figure 2 — Bifidobacterium intervention altered the gut microbiota in a DSS-induced mouse model. (A) Rarefaction curve of the estimated number of genera using the Chao1 index. (B) Principal coordinates analysis (PCoA) analysis. Each point in the figure represents a sample, and points of different colors indicate different groups. (C) Venn diagram of ASVs. Different colors represent different groups. (D) The bar graph and cladogram indicate the taxa that discriminate among the groups. Only taxa with an alpha value of 0.05 and with absolute LDA (log10) scores >2.0 were considered significant. (E) Taxonomic tree in packed circles. The largest circle represents the phylum level, and the gradually smaller circles represent class, order, family, genus, and species according to the gradient. The larger the sector area, the higher the abundance of the taxon in the corresponding group. (F) The ordinate is the relative abundance of the genus and the abscissa is the name of each sample in the grouping scheme. [file Image2.tif]

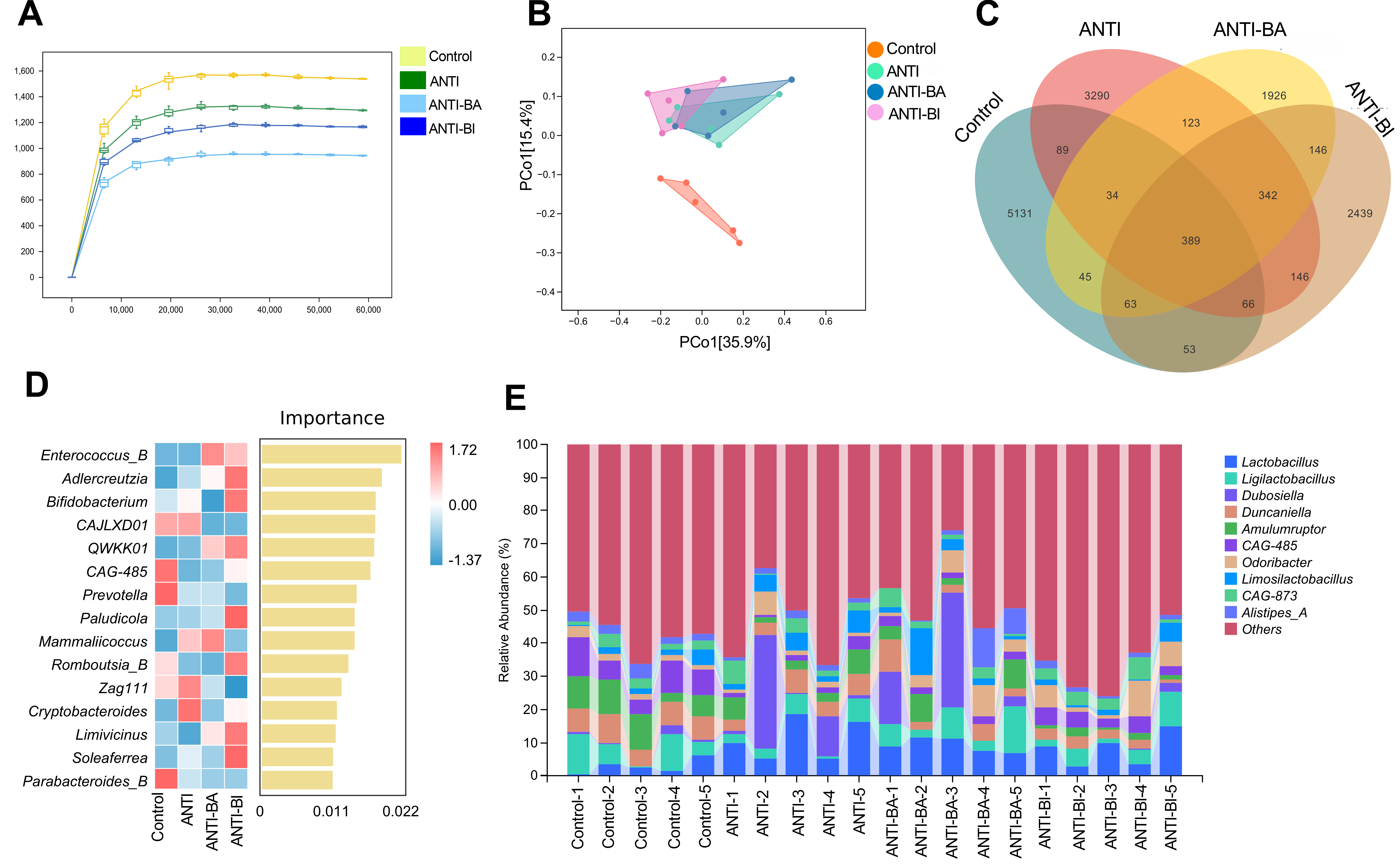

Supplement: Supplementary Figure 3 — Analysis of gut microbial diversity after BA and BI intervention based on 16S sequencing in a germ-free mouse model. (A) Rarefaction curve of the estimated number of genera using the Chao1 method. (B) PCoA analysis. Each data point represents an individual sample, with color coding distinguishing between different groups. (C) Venn diagram of ASVs. Different colors represent different groups. (D) Random Forest plot showing the 15 most predictive bacterial genera that differentiate different groups. (E) Relative Abundance in Genus. The y-axis represents the relative abundance of each genus, while the x-axis displays sample names, categorized according to the grouping scheme. [file Image3.tif]
